# Tuneable Liquid Crystal Micro-Lens Array for Image Contrast Enhancement in a Pixelated Thin Film Photo-Transistor Flat Panel Imager

**DOI:** 10.3390/mi8070205

**Published:** 2017-06-26

**Authors:** Kun Li, Daping Chu, Jiaqi Chu, Shuhei Kitajima, Tokiyoshi Matsuda, Mutsu Kimura

**Affiliations:** 1 Electrical Engineering Division, University of Cambridge, 9 JJ Thomson Avenue, Cambridge CB3 0FA, UK; kl330@cam.ac.uk (K.L.); jc818@cam.ac.uk (J.C.); 2 Department of Electronics and Informatics, Ryukoku University, Seta, Otsu 520-2194, Japan; t16m014@mail.ryukoku.ac.jp (S.K.); toki@rins.ryukoku.ac.jp (T.M.); mutsu@rins.ryukoku.ac.jp (M.K.)

**Keywords:** liquid crystal micro-lens, optical relay, thin film photo-transistor, image contrast

## Abstract

We propose and demonstrate the concept of using a tuneable liquid crystal micro-lens (LCML) array to improve the image contrast of a pixelated thin film photo-transistor (TFPT) flat panel imager. Such a device can be used to image contents on paper-based media and display a magnified version on a flat panel display for elderly or visually impaired people. Practical aspects including device physical geometry, object scattering profile, LC material, and focusing effect of LCML on an object are considered during the design process with the support of ZEMAX simulations. An optimised effective focal length (EFL) has been calculated for the designed LCML to best relay the objects or contents on a paper to the TFPT pixel plane. The designed LCML devices are fabricated with the optimised EFL, and they have good phase depth profiles which are close to a spherical lens profile. Preliminary test results show that the combination of a TFPT imager with an LCML array can make the image contrast more than two times better than that using the TFPT imager alone. The tuneable EFL of the developed LCMLs are useful in the situation where the LCML is not in direct contact with the imaged object.

## 1. Introduction

With the wide spread of flat panel displays (FPDs), people can choose to read contents of differently-sized fonts based on their preference and visual ability. Still, in the UK and US, a survey has revealed that majority of people (80% or more) prefer to read print on paper and believe that they understand much better and feel more relaxed compared to reading from screens [1,2]. Sadly, around 285 million people worldwide are visually impaired as estimated by the World Health Organisation in 2014, of which 65% are aged 50 and over [3]. In order for elderly and visually impaired people to read print on papers such as books, newspapers, and product tags/labels more efficiently, electronic magnifying viewers or video magnifiers have been developed [4]. They are better than conventional optical magnifiers because they can easily adjust brightness and contrast electronically to make the contents easier to read as well as have an option to choose from many levels of magnifications (e.g., from 1.5× to 50×) [5].

Handheld magnifiers have the features of being small, light, and portable, and they can achieve higher reading speed than their desktop counterparts (e.g., electronic vision enhancement systems) [6,7,8]. Conventional hand-held magnifying viewers use CCD cameras, and only a limited section of the contents can be imaged when the cameras are placed close to the paper media. Users have to move the viewer along the contents, and it is easy to lose track of where they are on the paper [9,10].

A high-resolution (254 ppi) active-matrix flat-panel imager has been demonstrated using poly-Si thin film photo-transistors (TFPTs), where an FPD is intended to be integrated to form a magnifying viewer [11,12]. The miniature trial product (16 × 16 pixels) of the flat-panel imager is fabricated on a glass substrate. The imager can scan letters of 1 × 1 mm^2^ font size. However, during the imaging process, the outline of the characters is blurry and image contrast between the background and characters is limited.

In this work, we propose the use of a tuneable liquid crystal micro-lens (LCML) array to improve imaging contrast of the TFPT flat panel imager. The LCMLs can be switched on and off, and their effective focal lengths (EFLs) can be varied by the driving voltages [13,14,15,16,17,18,19,20], which is not possible for the conventional fixed micro-lens arrays. Optical simulations are carried out using the ZEMAX software, and a designed LCML array is fabricated according to the designed device specifications and characterised with an optical microscope. Test results show the contrast improvement when LCML is in use.

## 2. Design Considerations

### 2.1. Consideration of the Practical Implementation and Design of a Micro-Lens Array

To implement a tuneable LCML on the TFPT imager in practice, four aspects are taken into consideration in the design of the LCML array. They include device physical geometry, object scattering profile, LC material, and focusing effect of LCML on an object.

**Figure 1:**
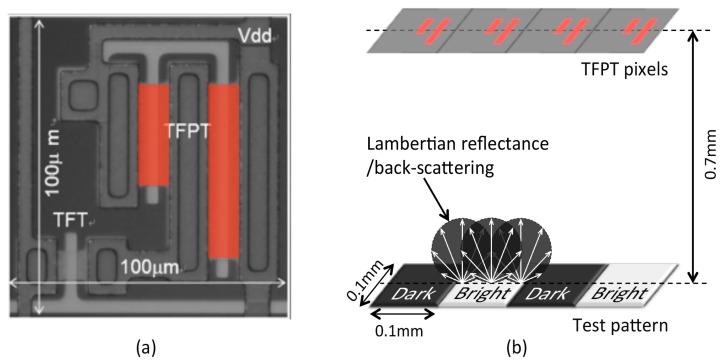
(**a**) Microscope image of a thin film photo-transistor (TFPT) pixel structure with the active part highlighted in red; (**b**) A sketch of the TFPT imager imaging an object with a Lambertian reflectance.

The device physical geometry includes the TFPT pixel dimension, distance from an object plane to the TFPT plane, and LCML device geometry. A microscope image of a TFPT pixel structure is shown in Figure 1a; it has a dimension of 0.1 × 0.1 mm^2^ and the active TFPT detection areas are highlighted in red. The TFPT pixel pitch is also 0.1 mm. An array of TFPT pixels is fabricated on a glass substrate of 0.7 mm in thickness, and this thickness determines the distance between an object plane and the TFPT plane. This distance contributes to a low image contrast because it is much larger than the pixel pitch. The glass thickness cannot be reduced, or the mechanical strength of the device will suffer.

In practical implementation, an object is a white paper (non-glossy) and it is widely regarded as an ideal diffusely reflecting surface because of its irregularities within the structure of the surface and surface roughness. As a result, our object has a Lambertian reflectance as shown in Figure 1b, which does not depend on incident angles of illuminating source. Together with the large distance between an object and the TFPT plane as well as the small TFPT pixel pitch, the TFPT imager will produce a lot of crosstalk between pixels and subsequently a low image contrast.

**Figure 2:**
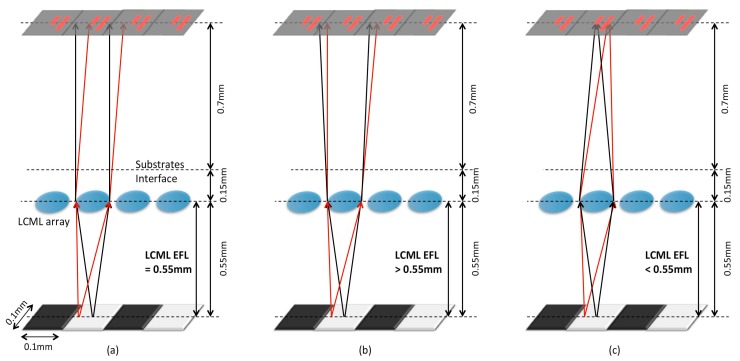
A sketch of a liquid crystal micro-lens (LCML) on the TFPT imager as the LCML effective focal lengths (EFL) is (**a**) equal to, (**b**) greater than, or (**c**) less than 0.55 mm.

When an LCML device is introduced in practice, each LCML corresponds to one TFPT pixel and its diameter must be ≤0.1 mm. Because there needs to be transparent electrodes—normally indium tin oxide (ITO)—between each LCML to electrically drive the LC, the LCML diameter is further reduced. The test LCML diameter is set to 0.07 mm due to the current settings of a laser machining system. An LC layer is sandwiched between two glass substrates; a thinner (0.15 mm) one is needed to minimise the overall distance between an object and the TFPT plane, and a thicker (0.55 mm) one is used as a mechanical support of the LCML device. A sketch of LCML on the TFPT implementation is shown in Figure 2, with the thicker, stronger glass substrate facing an object.

Three scenarios are shown in Figure 2a–c according to the LCML EFL range. When the LCML EFL is ≥0.55 mm, the light beam from a point on paper through the LCML are either collimated or less diverging, and they still land on the neighbouring TFPT pixels as shown in Figure 2a,b and result in a large crosstalk. However, with the LCML EFL < 0.55 mm, more subsequent light beams after the LCML tend to stay within the corresponding pixel and result in less crosstalk, as shown in Figure 2c. To satisfy the EFL range and 0.07 mm LCML diameter, a highly birefringent Nematic LC is recommended for a large phase variation, otherwise a thick layer of LC is needed which results in a long switch-off time. With an LC material of 0.275 birefringence at 550 nm wavelength at room temperature and a layer thickness of ≤10 µm, the fabricated LCML device can achieve EFL down to ~0.2 mm. Simulations are done to evaluate the EFL effect on image contrast, and results are discussed in the next section.

The LCML device has a focusing effect on a light beam from an illuminating source. A collimated light beam—assuming a light source is far away from an object compared to 0.55 mm—is focused onto an object’s surface, creating a hotspot if the LCML EFL is 0.55 mm. It is less important because firstly our paper-based object has a Lambertian reflectance. Secondly, the density of LCML is sufficiently high that hotspots are evenly distributed across an object’s surface.

### 2.2. Simulation Verification

**Figure 3:**
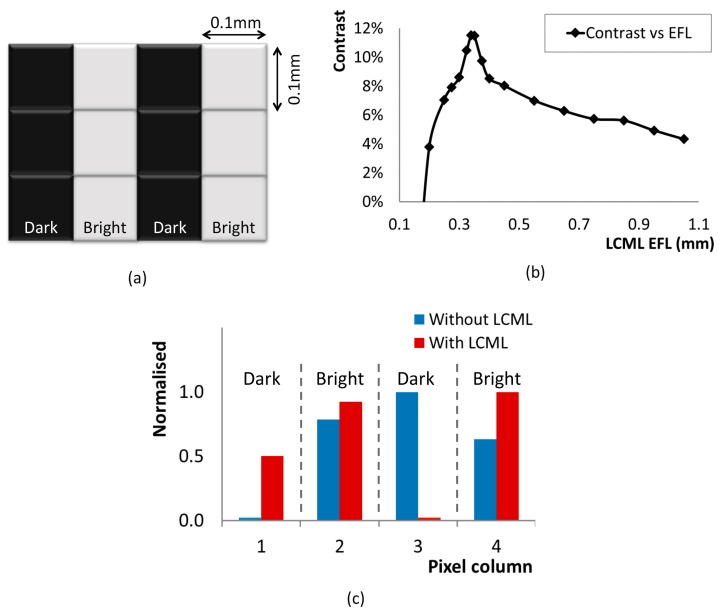
(**a**) Test object pattern of 4 × 3 square image pixels; (**b**) Simulated contrast with respect to varying LCML EFL; (**c**) Normalised light irradiance of the TFPT imager without and with an LCML array.

A ZEMAX simulation was set up with a test pattern of six bright and six dark square image pixels, each having a dimension of 0.1 × 0.1 mm^2^. The image pixels are arranged in a 4 × 3 array as shown in Figure 3a, forming a five line-pairs per millimetre (lp/mm) pattern. Each image pixel corresponds to one TFPT pixel. Bright image pixels are represented by Lambertian LED light sources, each with a total power of 1 W. A mask is placed directly in front of the TFPT pixel so that only the highlighted part of each TFPT pixel in Figure 1a is active in collecting light rays.

Without an LCML array, the TFPT plane is 0.7 mm away from the object plane, as shown in Figure 1b. With an LCML array, the TFPT plane is physically 1.4 mm away from the object plane, as shown in Figure 2. The LCML array, with a diameter of 0.07 mm, is represented with an array of spherical plano-convex lenses. Ray tracing was performed in ZEMAX with non-polarised light rays to determine light irradiance values (W/cm^2^) at each TFPT pixel. Irradiance is averaged at each column of TFPT pixels which correspond to either a column of dark or bright dots, respectively. These values are used to calculate image contrast via an equation $\left(\frac{I_{\max}-I_{\min}}{I_{\max}+I_{\min}}\right)\times 100\%$, where $I_{\max}$ is the maximum light irradiance of a pixel corresponding to the bright dots and $I_{\min}$ is the minimum light irradiance of a pixel corresponding to the dark dots. The LCML EFL can be varied from 0.1 to 1.1 mm to investigate its effect on image contrast, and the results are plotted in Figure 3b. Meanwhile, the simulated irradiance values are normalised according to an equation $\left(\frac{I-I_{\min}}{I_{\max}-I_{\min}}\right)$ and presented in Figure 3c for each TFPT imager column without and with LCML (highest contrast case), respectively.

The higher image contrast (>10%) appears when the LCML EFL is in the range of 0.32–0.37 mm. In this case, the object is being imaged onto the TFPT pixels through the LCML. At a lower EFL, the contrast drops dramatically until it reaches zero, where the TFPT imager cannot resolve a 5 lp/mm pattern. At a higher EFL, the contrast decreases steadily, as the LCML cannot effectively converge light rays from the object (bright dot) to the corresponding TFPT pixel.

Without an LCML array, the TFPT pixels have higher light irradiance because the TFPT is closer to the object. However, this is less important because the TFPT imager cannot resolve the line-pair pattern. The column three pixels corresponding to a column of dark dots have higher intensity values than ones corresponding to bright dots. As a result, the TFPT imager cannot produce a valid contrast value. With the LCML array (EFL = 0.34 mm), the TFPT pixels corresponding to dark dots generate lower values than ones corresponding to bright dots, as expected. In this case, the TFPT imager can resolve the line-pair pattern with a valid contrast of 11.5%.

## 3. Results

### 3.1. Device Fabrication and Characterisation

The LCML array used in this work consists of a proprietary nematic LC layer sandwiched between two display glass substrates, which were made in a cleanroom environment. The substrates are 0.15 and 0.55 mm thick with an ITO layer on the inner surfaces. The ITO coatings on the glass substrates were laser-machined to have an array of 16 × 16 circular through-holes of 0.07 mm in diameter, with a pitch of 0.1 mm. A homogeneous polyimide layer was spin coated and rubbed in an anti-parallel configuration on two substrates for the LC alignment.

**Figure 4:**
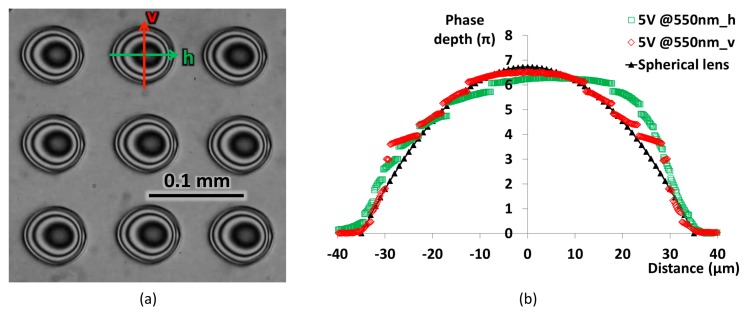
(**a**) Intensity variations of the LCML array under the microscope when a 5 V rms voltage signal is applied and (**b**) converted phase depth variation across the horizontal and vertical axis of one LCML.

The LC material has a birefringence of 0.275 at 550 nm wavelength at room temperature. We fabricated an LCML array with an LC thickness of 8 µm, which is theoretically able to provide a maximum phase delay of 8π. When a voltage signal (1 kHz square wave) is applied to the ITO layer, an electrical field gradient is formed at the machined hole rim, extending in a radial direction to the hole centre. LC directors will follow this field gradient and produce a spatial gradient of effective refractive index of the LC medium (i.e., a focusing lens) [20]. The phase depth variation inside the LCML can be calculated by measuring intensity changes under a microscope, as shown in Figure 4. The LCML device is arranged between cross-polarisers, with its rubbing direction at a 45° angle to the polarisation directions of both polarisers. The detailed measurement procedures and intensity–phase conversion are described in Ref. [18]. 

The intensity change in the LCML has a good symmetry in the vertical direction (v), and the converted phase depth variation shows a good match to a spherical lens profile, as shown in Figure 4b. In the horizontal direction (h), there is less symmetry in the intensity change; the bright and dark bands on the left half of the LCML are more spread than those on the right half, as shown in Figure 4a. As a result, the converted phase depth variation is more gradual on the left side and matches the spherical lens better than the right side, which has a sharp change between 20 and 35 µm. This non-symmetry in the phase profile is caused by the LC device configuration, especially an angle variation between the rubbing direction and the ITO hole rim, and it is beyond the scope of this study.

**Figure 5:**
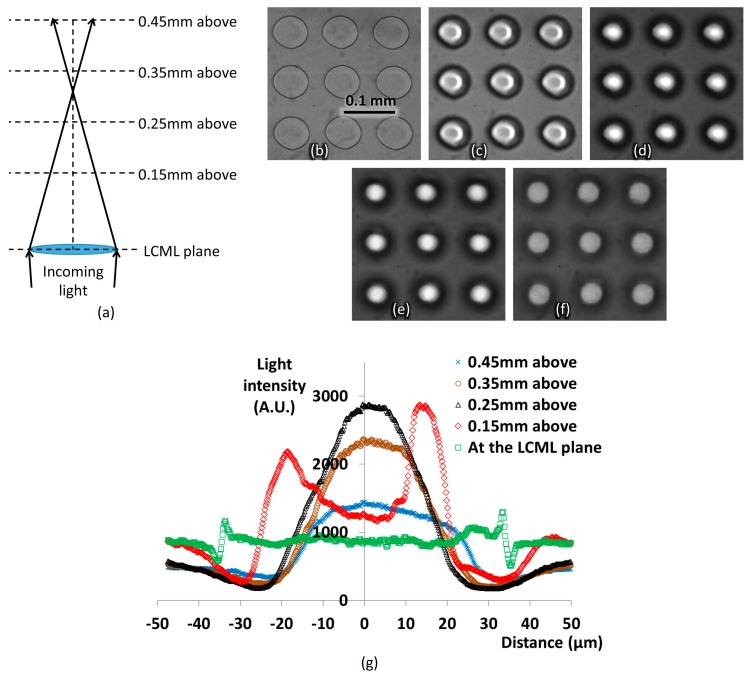
(**a**) A sketch of the microscope measurement setup. Images when the microscope is focused at (**b**) the LCML plane, (**c**) 150 µm above the LCML plane, (**d**) 250 µm above the LCML plane, (**e**) 350 µm above the LCML plane, and (**f**) 450 µm above the LCML plane. (**g**) Line profiles of light intensity across the centre of an LCML in the horizontal direction.

The EFL of the fabricated LCML array can be tuned from 0.3 to 0.85 mm by varying driving voltages between 4 and 10 V rms. The EFL is tested with the microscope in a transmission setup with a near-collimated light source (5° diverging angle) at 510 nm while the device is driven at 5 V rms, as shown in Figure 5a. The light source is polarised with its polarisation direction along the rubbing direction of the LCML array. Images at different planes are captured from the LCML plane to 0.45 mm above the LCML plane according to Figure 5a and shown in Figure 5b–f. An intensity line-profile is averaged over ~2 µm across the centre of LCML in the horizontal direction at each testing plane, and the results are shown in Figure 5g.

At the LCML plane, the light intensity is roughly the same across the interested region, shown as a uniform grey level in Figure 5b and its line-profile in Figure 5g. As the measurement moves towards 0.25 mm above the LCML plane, the region inside the LCML gets brighter because the light rays are converged to the centre after passing through the LCML. Moving continuously to 0.35 and 0.45 mm above the LCML plane, the peak intensity inside each micro-lens decreases; this is because the focal point of the LCML is passed as shown in Figure 5a. The highest light intensity is observed between 0.25 and 0.35 mm above the LC plane at 510 nm as shown in Figure 5g, which matches the designed EFL (0.34 mm) of the LCML at 550 nm.

To compare optical power distribution at different planes, light intensity is integrated over circles of 0.02 and 0.1 mm in diameter, which are at the same centre as the LCML. It is assumed that the light intensity distribution does not change when the profile-line rotates within the investigated plane at the LCML centre. At the LCML plane, about 4.3% of power is within the centre circle of 0.02 mm in diameter, which is similar to the ratio of areas between the 0.02 mm circle and 0.1 mm circle. At 250 and 350 µm above the LCML plane where the LCML EFL is, a 16–18% power is within the 0.02 mm centre circle, yielding a four-times increase due to the LCML focusing effect.

The line-profile is not a perfect symmetry because of the non-symmetrical phase profile in the horizontal direction, but most of the light is focused towards centre. This is sufficient for the application purpose of the TFPT imager because the contrast within each pixel is not taken into account.

### 3.2. Test on the TFPT Imager

**Figure 6:**
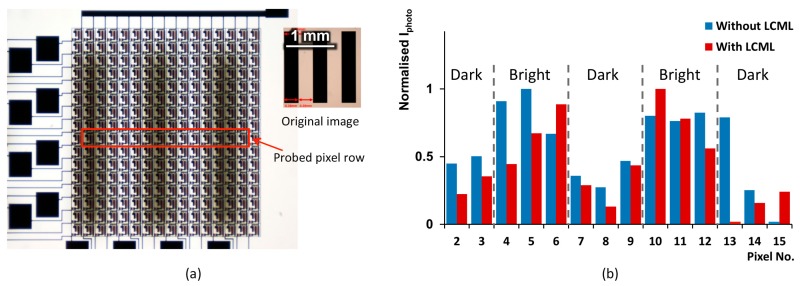
(**a**) A microscope image of the TFPT imager test setup with a line-pair test pattern of 1.8 lp/mm; (**b**) Normalised *I*_photo_ values of pixels 2–15 in row No. 9 without and with the LCML array.

The characterised LCML array is tested with the TFPT imager according to the setup in Figure 2c over a line-pair test pattern of ~1.8 lp/mm, shown as an inset (original) image in Figure 6a. When the TFPT plane is focused under the microscope, the test pattern becomes blurry. The photo-induced current (*I*_photo_) at the ninth pixel row is measured with a probe station. An un-polarised LED light source (2000 lx) is used to illuminate the test pattern for tests without the LCML array, and a polariser is placed at the exit of the light source for tests with the LCML array. The measured *I*_photo_ values are normalised and plotted in Figure 6b for tests without and with the LCML array; pixel Nos. 1 and 16 are not working properly, and their values are not presented.

When the LCML array is not used, *I*_photo_ values follow the original pattern and are higher at bright regions and lower at dark regions, apart from pixel No. 13. When the LCML array is used, there is a larger variation in *I*_photo_ across measured pixels due to the effect of image relay. *I*_photo_ values in dark regions are lower with the LCML than those without.

The measured data are processed (normalised and DC components removed) to calculate the image contrast. The contrast without the LCML is about 3.7% with a standard deviation of 1.6%; it is inconsistent between measurements. The contrast with the LCML (polarised light) is 8.4%—more than two times of value without the LCML—with a standard deviation of 1.1%. There are some discrepancies between the simulated and experimental values. The first is that the measured contrast with the LCML is not as high as the simulated one (11.5%). In the simulation, the LCML acts on all light rays, while in the measurement, the polariser does not have a perfect extinction ratio. Further, the back-scattering/reflectance of the object makes light even less polarised. As a result, a large number of light rays will not be focused by the LCML. In addition, the LCML does not have a perfect spherical phase profile. The second difference is that the TFPT can produce a valid contrast value without the LCML, compared with no contrast in the simulation. It is thought that the test pattern surface is not ideally diffusing and it has a much smaller diffusing angle compared with the Lambertian surface.

## 4. Discussion and Future Work

LCML has an important property of being able to tune its EFL by varying the driving voltage levels in a reasonable range. In our case, the EFL was changed from 0.3 to 0.85 mm with 4–10 V rms. A larger range of EFL tuning can be achieved by changing an LCML device design if needed. EFL tuning is essential because we cannot always be sure that the glass substrate of LCML is in close contact with an object without any gap. For example, a protection layer of some sort or dusts and dirt on the surface will all increase the distance between an object and an LCML array. In such cases, the LCML EFL needs to be increased accordingly in order to produce a clear image with the maximum image contrast.

LCML has a reasonably short response time, and is sufficient for the TFPT imager application. The switch-off time from LC on to LC off for the fabricated device is proportional to the square of the LC layer thickness for the same material. In our case, it is in the expected range of 80–100 ms with an 8 μm-thick LC layer when driven by 10 V rms at room temperature. The switch-on time from LC off to LC on is faster—in the range of 20–40 ms by the same driving voltage. It can be much faster—in a few ms—if overdriving scheme is used.

The use of LCML on the TFPT imager is effective and can also be scaled up. The existing LC display assembly line can be easily adapted to fabricate the LCML array device, with an additional step to create through-holes in the ITO coating for the LCML array. The machining of the ITO layer can be achieved with a laser scanning system, which can scan up to 10 m/s. As a result, it only takes ~20 s to create two million holes/LCMLs. Such a device can match a full HD (1920 × 1080) TFPT imager with a dimension of 192 × 108 mm^2^, which is sufficiently large for a hand-held video magnifier.

Further improvement can be made to a LCML array. In this study, the diameter of the LCML is 0.07 mm due to the laser system setup. The diameter can be potentially increased to 0.09 mm to increase the contrast further. ZEMAX simulation suggests that by using the 0.09 mm diameter LCML array, the contrast can reach 27% compared with 11.5% for the 0.07 mm diameter. In addition, the LCML array can be built directly on the TFPT glass substrate to eliminate the 0.15 mm glass in-between. In this case, a slightly larger contrast of 14.5% can be achieved (in simulation) with the 0.07 mm diameter LCML. It is clear that a larger LCML diameter is more effective in achieving higher contrast.

## 5. Conclusions

A tuneable LCML array was designed, fabricated, and characterised. It can be used with a TFPT flat panel imager for the improvement of the recorded image contrast of a hand-held magnifier as designed for visually impaired people. Practical aspects including device physical geometry, object scattering profile, LC material, and focusing effect of LCML on an object are taken into consideration during the design process with help of ZEMAX simulations. It is calculated that with an optimised EFL of 0.34 mm, the designed LCML array can relay objects or contents on a paper to the TFPT pixels and the imager yields a value of 11.5% when imaging a Lambertian surface. The designed LCML arrays can be manufactured with an existing LC display assembly line and an additional laser machining process that is capable of large-scale production. The fabricated LCML array was tested experimentally on its own with a good light focus ability. When tested together with a TFPT imager, it showed that the contrast of the recorded images increased by more than twice. Furthermore, the tuneable EFL of the developed LCML array can be used to relay the image when the imaged object is away from the LCML array.
